# Similar risk of complete revision for infection with single-dose versus multiple-dose antibiotic prophylaxis in primary arthroplasty of the hip and knee: results of an observational cohort study in the Dutch Arthroplasty Register in 242,179 patients

**DOI:** 10.1080/17453674.2020.1794096

**Published:** 2020-07-23

**Authors:** Ewout S Veltman, Erik Lenguerrand, Dirk Jan F Moojen, Michael R Whitehouse, Rob G H H Nelissen, Ashley W Blom, Rudolf W Poolman

**Affiliations:** a Department of Orthopaedic and Trauma Surgery, Joint Research, OLVG, Amsterdam, the Netherlands; b Department of Orthopaedics, Leiden University Medical Center, Leiden, the Netherlands; ^3^Musculoskeletal Research Unit, Translational Health Sciences, Bristol Medical School, University of Bristol, Bristol, UK; d National Institute for Health Research Bristol Biomedical Research Centre, University Hospital Bristol NHS Foundation Trust and University of Bristol, UK

## Abstract

Background and purpose — The optimal type and duration of antibiotic prophylaxis for primary arthroplasty of the hip and knee are subject to debate. We compared the risk of complete revision (obtained by a 1- or 2-stage procedure) for periprosthetic joint infection (PJI) after primary total hip or knee arthroplasty between patients receiving a single dose of prophylactic antibiotics and patients receiving multiple doses of antibiotics for prevention of PJI.

Patients and methods — A cohort of 130,712 primary total hip and 111,467 knee arthroplasties performed between 2011 and 2015 in the Netherlands was analyzed. We linked data from the Dutch arthroplasty register to a survey collected across all Dutch institutions on hospital-level antibiotic prophylaxis policy. We used restricted cubic spline Poisson models adjusted for hospital clustering to compare the risk of revision for infection according to type and duration of antibiotic prophylaxis received.

Results — For total hip arthroplasties, the rates of revision for infection were 31/10,000 person-years (95% CI 28–35), 39 (25–59), and 23 (15–34) in the groups that received multiple doses of cefazolin, multiple doses of cefuroxime, and a single dose of cefazolin, respectively. The rates for knee arthroplasties were 27/10,000 person-years (95% CI 24–31), 40 (24–62), and 24 (16–36). Similar risk of complete revision for infection among antibiotic prophylaxis regimens was found when adjusting for confounders.

Interpretation — In a large observational cohort we found no apparent association between the type or duration of antibiotic prophylaxis and the risk of complete revision for infection. This does question whether there is any advantage to the use of prolonged antibiotic prophylaxis beyond a single dose.

Annually around 1 million patients receive a total hip or total knee prosthesis in the United States and over 190,000 hip and knee replacements are performed in England and Wales (Maradit et al. 2015, National Joint Registry for England and Wales 2018). The incidences of prosthetic replacement of the hip and knee are expected to increase (Kurtz et al. 2014). Prosthetic joint infection (PJI) following total hip or knee arthroplasty and the treatment thereof are catastrophic for patients and pose tremendous costs to healthcare systems (Poultsides et al. 2010, Zmistowski et al. 2013, Moore et al. 2015). Perioperative antibiotic prophylaxis remains an effective method of reducing the risk of PJI (Illingworth et al. 2013, Thornley et al. 2015). The type and duration of antibiotic prophylaxis are subject to debate.

Both single-dose and multiple-dose antibiotic prophylaxis regimens have been advocated with comparable results (Thornley et al. 2015, Tan et al. 2019). The recommendations provided by the Second International Consensus Meeting of the MusculoSkeletal Infection Society (MSIS) and the European Bone and Joint Infection Society (EBJIS) advise that antibiotic prophylaxis should be administered 30–60 minutes before incision and discontinued within 24 hours after surgery (Hansen et al. 2014, Parvizi and Gehrke 2018). Large variation in prophylaxis regimens has been observed in the United Kingdom (Hickson et al. 2015). The Dutch national orthopedic association advises administration of antibiotic prophylaxis using a first- or second-generation cephalosporin starting 30–60 minutes preoperatively and discontinuing the antibiotic prophylaxis within 24 hours (Swierstra et al. 2009, Nederlandse Orthopaedische Vereniging 2018). The World Health Organization and, in the USA, the Centers for Disease Control and Prevention (CDC) recommend against the use of postoperative continuation of antibiotic prophylaxis and advocate for a single dose of antibiotics delivered preoperatively (Berrios-Torres et al. 2017). This recommendation is vehemently challenged by the American Association of Hip and Knee Surgeons and the International Consensus Meeting, which encourage their members to proceed with the current common practice of multiple-dose antibiotic prophylaxis protocols until more evidence is available (Yates 2018).

We compared the risk of complete revision for infection in the 1st year following primary hip and knee arthroplasty according to the perioperatively administered antibiotic prophylaxis regimen by using data from the Dutch Arthroplasty Register (LROI).

## Patients and methods

This study was structured using the STROBE guideline. In this observational cohort study, we report analyses of data for the Netherlands from the Dutch Arthroplasty Register (LROI) between January 1, 2011 and December 31, 2015. We included in the study all patients who had a primary hip or knee replacement during this period. Patient consent was obtained for data collection and linkage by the LROI. Using data on patient level was not possible due to the legislation of the General Data Protection Regulation.

In the absence of individual patient-level data on antibiotic prophylaxis, we performed a national audit of hospital perioperative antibiotic prophylaxis regimens in the Netherlands (Veltman et al. 2018). All 99 Dutch hospitals or clinics performing primary total hip arthroplasty (THA) or total knee arthroplasty (TKA) were contacted and all completed a survey to identify the existence of treatment protocols concerning primary joint replacement, the existence of protocols regarding treatment strategy in case of suspected early postoperative infection, and tendency to register procedures in the LROI database. We asked, in particular, about type and duration of antibiotic prophylaxis. This survey showed a variance in postoperative duration of antibiotic prophylaxis. 10 Dutch hospitals administered a single-shot antibiotic prophylaxis, while the remaining 89 administered a multiple-shot antibiotic prophylaxis. This variance facilitated an observational cohort study using the LROI. The LROI has a completeness of over 95% for primary hip and knee arthroplasties and of 91% and 92% for the hip and knee revision procedures respectively (Dutch Arthroplasty Register [LROI] 2014, 2017, van Steenbergen et al. 2015). The translated survey form can be found in Appendix 1, Supplementary data.

Each patient who had a primary THA or TKA was followed up for a minimum of 12 months until the end of the observation period (December 31, 2015) or until the date of 1- or 2-stage revision for infection, revision for another indication, death or end of follow-up (January 1, 2018). Revisions for infection included only complete revision of the total system, obtained by a 1- or 2-stage revision procedures. All partial revisions (e.g., debridement, antibiotics, and implant retention procedures [DAIR]) were excluded because these partial revisions are inconsistently recorded compared with total revisions (Dutch Arthroplasty Register [LROI] 2017, Veltman et al. 2018). We chose to end the follow-up period at 1 year after surgery as with longer follow-up the influence of hematogenous infections on the measured outcome may increase to become larger than the influence of the duration of antibiotic prophylaxis at primary surgery.

We defined infection status using the surgical indication reported on the LROI revision arthroplasty form following surgery by the treating orthopedic surgeon. We included patients whom had undergone complete revision captured by the LROI where the reason for revision was defined as infection in the infected group and patients in whom the reason for revision was not reported, or reason for revision other than infection was reported, in the non-infected group. The diagnosis and treatment strategy for complete revision for infection was at the discretion of the surgeon and treating unit and it reflected contemporary practice over the study period, with raised inflammatory markers, joint-specific symptoms, sinuses, and positive microbiological cultures being common diagnostic features over that period (Parvizi et al. 2013).

We compared the risk of complete revision surgery for infection in the 1st year following primary arthroplasty by the type and duration of antibiotic prophylaxis regimen administered at primary surgery. We considered the patient characteristics age, sex, BMI, ASA grade, and previous surgery. We considered surgical factors such as indication for surgery, surgical approach, type of fixation, and bearing surface. Data from the LROI database were combined at hospital level with the results of the national survey on antibiotic prophylaxis. Results of the survey show there were 3 types of antibiotic regimens that are used in the Netherlands: multiple doses of cefazolin (MCZ), multiple doses of cefuroxime (MCX), and single dose of cefazolin (SCZ), which are all in concordance with the Dutch guideline for perioperative antibiotics in total hip and knee arthroplasty (Veltman et al. 2018). No other antibiotic regimens were encountered in the survey. Patients were divided into 3 groups (MCZ, MCX, and SCZ) according to the antibiotic prophylaxis protocol of the hospital where they were treated.

### Statistics

We investigated the association between hospital antibiotic prophylaxis regimen policies (MCZ used as the reference) and the risk of complete revision for infection in the first 12 months following the index primary surgery with Poisson regression to account for time at risk and to produce hazard ratios including 95% confidence intervals (CI). The baseline hazard rate was modelled with restricted cubic splines. The optimum numbers of knots (3 degrees of freedom [d.f.] for the hip models, 4 d.f. for the knee models) was identified with AIC and BIC criteria (Appendix Table 1, Supplementary data). Interaction terms between the splines and the main exposure covariates were included to estimate the time-dependent hazard ratio for complete revision for infection of the different antibiotic prophylaxis regimens (Royston and Lambert 2011). Huber–White sandwich estimates of variance were computed to adjust for within-hospital correlation. The models were stratified by surgical site and adjusted for age, sex, BMI, and ASA classification. Multiple imputation by chained equations (5 imputations sets) under a missing at random framework was used to account for missing data. The imputation model incorporated the PJI status, time at risk, the main exposure, the aforementioned adjustment factors and indication for surgery, surgical approach, method of fixation, bearing surface, and year of surgery as ancillary variables. All statistical analyses were performed using Stata, version 15.1 (StataCorp, College Station, TX, USA).

### Ethics, registration, funding, and potential conflicts of interest

This study was approved by the scientific committee of the LROI. The database was constructed by the LROI office. All data provided by the LROI were anonymized, no patient identifiable data were available to the researchers.

The study protocol was registered on ClinicalTrials.gov (reference NCT03348254).

This study was partially supported by the NIHR Biomedical Research Centre at University Hospitals Bristol NHS Foundation Trust and the University of Bristol. The views expressed in this publication are those of the author(s) and not necessarily those of the NHS, the National Institute for Health Research, or the Department of Health and Social Care.

The National Institute for Health Research had no role in study design, data collection analysis, interpretation, or writing of the report. The corresponding author had full access to all the data in the study and had final responsibility for the decision to submit for publication. The authors have no conflicts of interest to declare.

## Results

During 2011 to 2015, 130,712 primary total hip arthroplasties and 111,467 primary total knee arthroplasties were performed across 99 centers. 399 hips and 303 knees were revised within 1 year of the primary arthroplasty for an indication of infection (Tables 2 and 3, see Supplementary data). Multiple-dose cefazolin (MCZ), multiple-dose cefuroxime (MCX), or single-dose cefazolin (SCZ) antibiotic prophylaxes were respectively administrated to 87%, 4%, and 9% of patients. Hereafter, “revision” refers to “1 and 2-stage revisions.”

**Figure 1.**
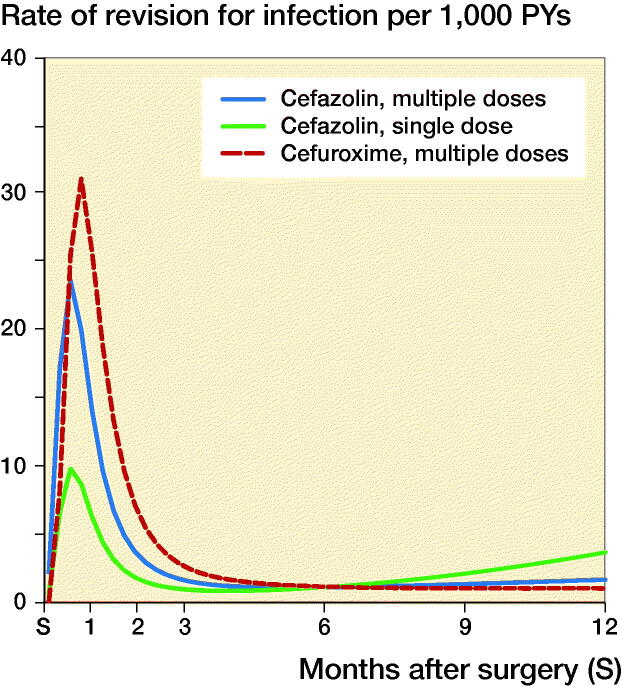
Rate of complete revision for infection in the first 12 months following primary hip replacement by type of antibiotics regimen.

**Figure 2.**
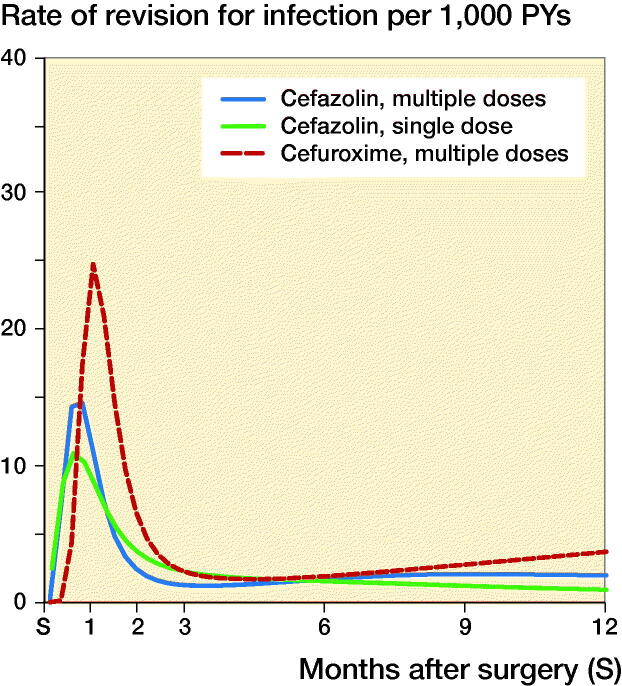
Rate of complete revision for infection in the first 12 months following primary knee replacement by type of antibiotics regimen.

For total hip arthroplasties, the 1-year rates of revision for infection (CI) were respectively 31/10,000 person-years (28–35), 39 (25–59), and 23 (15–34) in the groups that received MCZ, MCX, and SCZ; the rates for knee arthroplasties were 27 (24–31), 40 (24–62), and 24 (16–36) respectively. The rates of revision for infection over time according to antibiotic prophylaxis regimen are shown in Figures 1 and 2. Revision for infection was performed most frequently in the first 3 months postoperatively for both hip and knee replacements.

**Figure 3.**
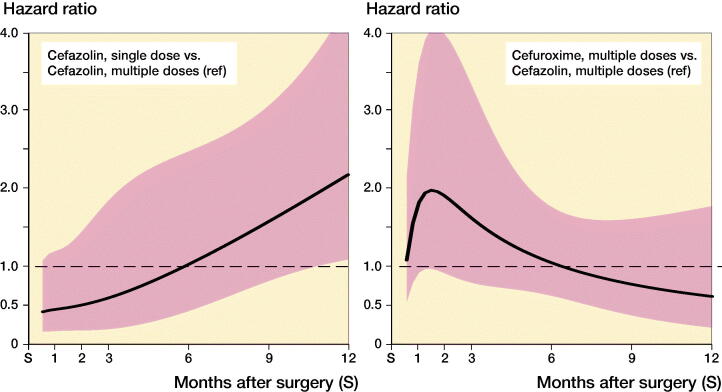
Hazard ratio and 95% CI* of complete revision for infection in the first 12 months following primary hip replacement by type of antibiotics regimen (reference: cefazolin multiple dose). * Derived from unadjusted Poisson model with restricted cubic splines (3 degree of freedom) (see Appendix Table 2).

**Figure 4.**
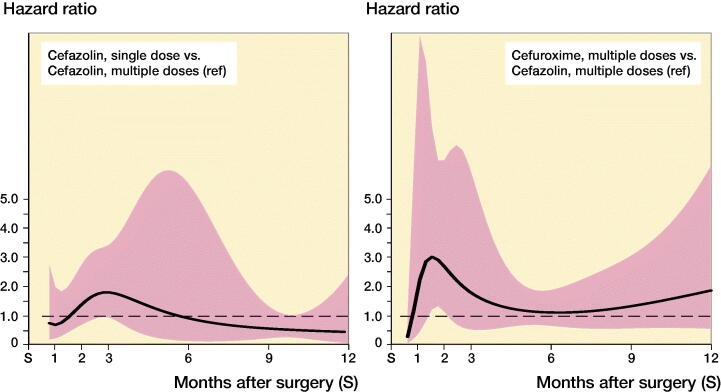
Hazard ratio and 95% CI* of complete revision for infection during the first 12 months following primary knee replacement by type of antibiotics regimen (reference: cefazolin multiple dose). *Derived from unadjusted Poisson model with restricted cubic splines (3 degree of freedom) (see Appendix Table 3).

While the risk of complete revision for infection appeared to differ over time, no or little evidence of differences between antibiotic prophylaxis regimens was found (Figures 3 and 4). In the first 11 months after primary hip arthroplasty, the risk of revision was comparable between SCZ and MCZ (adjusted HR SCZ vs. MCZ at 3 months 0.59 [0.19–1.8], at 6 months 1.02 [0.43–2.4]), but the risk of revision was higher in the SCZ group thereafter (HR 2.2 [1.1–4.4]). No evidence of difference was found between MCZ and MCX following hip arthroplasty (adjusted HR MCX vs. MCZ at 3 months 1.5 [0.77–3.1], at 6 months 1.0 [0.60–1.7], at 12 months 0.61 [0.20–1.8]). For patients receiving a primary total knee arthroplasty revision rates between SCZ and MCZ were comparable (adjusted HR SCZ vs. MCZ at 3 months 1.8 [0.87–3.8], at 6 months 0.89 [0.15–5.3], at 12 months 0.47 [0.09–2.4]). The risk of revision for infection was also comparable between MCZ and MCX (adjusted HR MCX vs. MCZ at 3 months 1.7 [0.54–5.4], at 6 months 1.2 [0.65–2.0], at 12 months 1.9 [0.56–6.1]). The patterns observed were comparable in the unadjusted and adjusted models (Tables 1 and 2).

**Table 1.** Hazard ratio (HR) of complete revision for infection in the first 12 months following primary hip replacement (reference: cefazolin multiple dose)

Months from primary procedure	cefazoline single dose HR (95% CI)	cefuroxime multiple dose HR (95% CI)
Unadjusted HR		
1	0.45 (0.17–1.20)	1.82 (0.92–3.62)
2	0.50 (0.17–1.42)	1.92 (0.92–4.01)
3	0.60 (0.19–1.87)	1.59 (0.78–3.25)
6	1.04 (0.43–2.49)	1.03 (0.61–1.74)
9	1.59 (0.82–3.09)	0.76 (0.36–1.61)
12	2.18 (1.09–4.38)	0.61 (0.21–1.78)
Adjusted HR **^a^**		
1	0.45 (0.17–1.20)	1.80 (0.92–3.52)
2	0.49 (0.17–1.38)	1.88 (0.92–3.86)
3	0.59 (0.19–1.79)	1.54 (0.77–3.08)
6	1.02 (0.43–2.39)	1.00 (0.60–1.68)
9	1.59 (0.83–3.02)	0.75 (0.35–1.61)
12	2.21 (1.12–4.38)	0.61 (0.20–1.81)

**^a^**Adjusted for age, sex, BMI, and ASA grade.

**Table 2.** Hazard ratio (HR) of complete revision for infection in the first 12 months following primary knee replacement (reference: cefazolin multiple dose)

Months from primary procedure	cefazoline single dose HR (95% CI)	cefuroxime multiple dose HR (95% CI)
Unadjusted HR		
1	0.78 (0.33–1.84)	2.24 (0.48–10.5)
2	1.52 (0.78–2.95)	2.70 (1.15–6.30)
3	1.77 (0.86–3.63)	1.72 (0.54–5.50)
6	0.89 (0.15–5.26)	1.13 (0.66–1.91)
9	0.58 (0.26–1.26)	1.36 (0.59–3.11)
12	0.47 (0.09–2.40)	1.88 (0.58–6.10)
Adjusted HR **^a^**		
1	0.78 (0.33–1.83)	2.34 (0.49–11.2)
2	1.55 (0.80–3.02)	2.70 (1.16–6.29)
3	1.81 (0.87–3.76)	1.71 (0.54–5.37)
6	0.89 (0.15–5.31)	1.15 (0.65–2.02)
9	0.58 (0.26–1.28)	1.38 (0.58–3.30)
12	0.47 (0.09–2.37)	1.88 (0.56–6.31)

**^a^**Adjusted for age, sex, BMI, and ASA grade

## Discussion

In this large observational cohort study of primary total hip and knee replacement, our findings suggest a comparable risk of complete revision for infection between the antibiotic prophylaxis regimens in terms of type of antibiotic and duration of prophylaxis during the first 12 months following surgery. When examining the hazard ratios, it is important to note that the majority of infections occurred within the first 3 months of surgery. Comparing single and multi-dose prophylaxis with cefazolin for hip replacement, the hazard ratio for complete revision for infection following single-dose prophylaxis steadily increased over time from less than half of that with multi-dose to over double the incidence of infection by month 12. This may be due to low-virulence micro-organisms that are more susceptible to multi-dose therapy presenting with infection later. If this is true, the differences between the different regimes should become more apparent with longer follow-up. This was not the case following knee replacement and alternatively may simply reflect either a chance occurrence, differences in patient- and surgery-related factors, or residual confounding. Adjustment for established confounding variables (age, sex, BMI, ASA grade) did not change these results.

We observed that the highest risk of complete revision for infection in the year following surgery occurred within the first 3 months after the operation. Rates then appear to rise again towards the end of the follow-up period. These patterns are consistent with contemporary patterns found in other registries (Dale et al. 2012, Lenguerrand et al. 2017a, 2017b). This may be due to the effect of more virulent microorganisms presenting during the first 3 months and less virulent microorganisms presenting later. Since the LROI does not provide data on which microorganism is causing the PJI, this remains speculative. Another reason might be a genuine increase in the incidence of PJI or may reflect more rapid diagnosis and aggressive treatment of PJI in recent years. We have not analyzed procedures where only debridement or partial revision (including debridement and implant retention [DAIR] with modular exchanges) were performed as these procedures are not reliably captured by the LROI registry (Veltman et al. 2018). DAIR has been shown to treat infection effectively in approximately 46–76% of cases (Wouthuyzen-Bakker et al. 2020). We have no reason to believe that the use of DAIR is related to type or duration of antibiotic prophylaxis, but it is a possible cause of residual confounding.

It has been suggested that the most appropriate perioperative prophylactic antibiotic is a first- or second-generation cephalosporin (i.e., cefazolin or cefuroxime) administered intravenously within 30 to 60 minutes prior to incision as a single and weight-adjusted dose (AlBuhairan et al. 2008, Stefansdottir et al. 2009, Steinberg et al. 2009). This policy is part of antibiotic stewardship, performed in countries with a low prevalence of MRSA (Illingworth et al. 2013, American Academy of Orthopaedic Surgeons/American Association of Orthopaedic Surgeons 2014). While consensus exists on type of antibiotic prophylaxis (Parvizi and Gehrke 2018) the postoperative duration of antibiotic prophylaxis remains subject to discussion.

A recent systematic review and meta-analysis by Thornley et al. (2015) explored whether or not a single preoperative antibiotic dose is adequate for arthroplasty patients. The review included 4 RCTs including 4,036 patients (Heydemann and Nelson 1986, Ritter et al. 1989, Wymenga et al. 1992, Kanellakopoulou et al. 2009). They concluded that additional postoperative antibiotic doses did not reduce the rates of infections (3.1% versus 2.3% postoperative PJI for multiple-dose and single-dose prophylaxis respectively). However, they reported that the quality of the included studies was very low. 3 of these studies were performed more than 20 years ago, while the other study used teicoplanin, which is no longer recommended for use as antibiotic prophylaxis (Berrios-Torres et al. 2017). Heydemann and Nelson (1986) randomized 211 patients between single-dose and 48-hour multiple-dose prophylaxis, but found no cases of PJI in either group. Ritter et al. (1989) compared a single dose of cefuroxime to 24 hours of postoperative prophylaxis in 196 patients, and found no cases of PJI in either group. Wymenga et al. (1992) randomized 3,013 patients in a multicenter RCT comparing a single preoperative dose of cefuroxime to a group receiving three doses and found no significant differences in PJI rates between groups. Engesaeter et al. (2003) reported the lowest rate of infection for patients who received 4 doses of antibiotic prophylaxis in 24 hours, compared with patients who received 1, 2, or 3 doses in their study of the Norwegian Arthroplasty Register. All authors of these studies recognized their study sample to be underpowered for determining a difference in PJI rates and recommended further studies to provide a definite answer. Based on these studies, the CDC has recently recommended against the use of postoperative continuation of antibiotic prophylaxis (Berrios-Torres et al. 2017). The recent International Consensus meeting advises to continue antibiotics postoperatively for 24 hours until better quality evidence is available (Parvizi and Gehrke 2018). A protocol for an RCT randomizing patients receiving a total knee arthroplasty between single-dose versus multiple-dose antibiotic prophylaxis has been registered on clinicaltrials.gov (NCT03283878). The study aims to answer definitively what duration of antibiotic prophylaxis is best. However, the planned follow-up of 90 days seems too short to capture all relevant infections. Also, the sample size is not justified in the trial registration, but with the aim of including 8,000 patients the study seems underpowered.

Our study has several strengths. The large numbers studied allows adequate power to detect rare outcomes such as complete revision for infection. Data capture represents over 98% of national activity (Dutch Arthroplasty Register 2017). This rate of coverage provides excellent external validity and generalizability of our findings. The rate of complete revision for infection within 1 year of primary arthroplasty is higher for males, patients with higher BMI, or higher ASA grade in all groups, independent of the type of antibiotic prophylaxis (Dale et al. 2012, Lenguerrand et al. 2018). This is in agreement with the literature and highlights the comparability of this Dutch arthroplasty cohort to other studied cohorts (Dale et al. 2012, Lenguerrand et al. 2018, Kunutsor et al. 2018b).

In order to establish the current practice for antibiotic prophylaxis regimes, we conducted a comprehensive national survey to determine current practice. The outcome of interest is a binary endpoint, and whilst this may mean that not all cases of PJI are captured, as many may be treated without complete revision surgery, it does make the end-point easily defined (Blom et al. 2003). In the absence of randomized controlled trials on the type and duration of antibiotic prophylaxis, this natural experiment in a large and generalizable national registry represents the best data currently available to determine whether there is a difference in the risk of complete revision for infection according to the antibiotic prophylaxis regimen.

The study does have limitations. The LROI database was established as an arthroplasty register, and whilst one of the outcomes of interest is complete revision for infection, the register was not designed to capture all infection outcomes and thus there is likely to be underreporting of infection as may also be the case in other national arthroplasty registries (Gundtoft et al. 2015, Kunutsor et al. 2018b). The most notable effect of this is the lack of capture of further procedures performed after the primary surgery to manage infection, such as DAIR procedures. The Dutch survey showed only 64% of hospitals registered DAIR procedures in the LROI, thus we did not include these in our analysis. As about 50% of PJI may be treated only with DAIR and arthroplasty registries are known to provide an underestimation of the rate of prosthetic revisions due to PJI of 20%, we may be missing as much as 70% of all treated infections (Gundtoft et al. 2015, Kunutsor et al. 2018a). Although prospectively collected our data are observational, and we can only draw conclusions on the nature and magnitude of the associations but cannot establish causative relation due to the possibility of residual confounding and estimation uncertainty. Whilst we conducted a comprehensive survey to establish the current practice in terms of antibiotic prophylaxis regimes, it is likely that for various reasons, including allergy, intolerance, and surgeons’ preference, not all patients received the antibiotics as per hospital protocol. However, a recent large retrospective study in the USA showed that 95% of patients received standard antibiotic prophylaxis (Wyles et al. 2019). The three types of antibiotics all are cephalosporins with the same allergy profile, therefore the percentage of patients with allergies should be comparable in all groups. Changes to the local antibiotic protocols during the study period have not been captured by the survey. The Dutch guideline for antibiotic prophylaxis around primary hip and knee arthroplasty did not change during the time period. However, changes to the antibiotic protocols may have occurred between the groups in all directions. Due to the quasi-randomized allocation of our patients, this should not introduce systematic bias.

Thus, this study resembles a natural experiment. Rather than controlling for observed confounders and expecting no unobserved confounders to be present (as in multiple regression, matching, and reweighting), natural experiments identify variation in the exposure, known to be independent of other confounders (Bor 2016). In our study quasi-random variation in the exposure (antibiotic prophylaxis regimen after total hip or knee arthroplasty) arises from naturally occurring random variation due to allocation of patients to the regional hospital near their residence. Natural experiments minimize the risk of confounding due to selective exposure to the intervention or residual confounding, and have internal validity and transparency of assumptions (Bor 2016). To establish true causality, a superiority or non-inferiority randomized controlled trial is still needed. However, as PJI is rare, the numbers needed for such a trial would be very large. Nonetheless, as the impact of PJI is so devastating (Moore et al. 2015) we recommend that such a trial is undertaken and suggest that embedding such a trial in a national arthroplasty registry may reduce costs and improve feasibility. Until such time, the data represented here are the best available evidence and it must be questioned whether there is any advantage to the use of prolonged antibiotic prophylaxis beyond a single dose.

## Supplementary Material

Supplemental Material

## Supplementary data

The Appendix Tables 1–3 are available as supplementary data in the online version of this article, http://dx.doi.org/10.1080/17453674.2020.1794096

The authors thank the patients and staff of all the hospitals who have contributed data to the LROI database. They are grateful to the Netherlands Orthopaedic Association (NOV) and to the LROI for granting access to this database. We thank Liza van Steenbergen of the LROI office for her help with data extraction and her prompt support with our data management queries.
