# Supplementary material for: Similar risk of complete revision for infection with single-dose versus multiple-dose antibiotic prophylaxis in primary arthroplasty of the hip and knee: results of an observational cohort study in the Dutch Arthroplasty Register in 242,179 patients
Source: Acta Orthop. 2020 Jul 23;91(6):794–800. doi: 10.1080/17453674.2020.1794096 (PMC8023957; doi:10.1080/17453674.2020.1794096)
Supplement: Supplemental Material [file IORT_A_1794096_SM7241.pdf]

Supplementary data

Appendix Table 1. Model fit

Model	d.f. [a]	AIC [b]	BIC [c]
Hip	2	2570	2586
	3 [d]	2429	2447
	4	2431	2452
	5	2432	2456
Knee	2	2218	2234
	3	2133	2151
	4 [d]	2111	2132
	5	2114	2139

[a] Degrees of freedom.
[b] Akaike information criterion.
[c] Bayesian information criterion.
[d] The models that minimized the AIC and BIC criteria were selected to identify the number of optimal knots for the spline function (number of degrees of freedom—1). The log of follow-up time was modelled to obtain better fitting models.

Appendix Table 2. Revision rates for infection per 10,000 person-years (PYs) after hip replacement by antibiotics regimen

	Cefazolin multi-dose				Cefazolin single -dose				Cefuroxime multi-dose			
Factor	Revisions	n	PYs	Rate (95% CI)	Revisions	n	PYs	Rate (95% CI)	Revisions	n	PYs	Rate (95% CI)
Overall	350	113,285	111,562	31.4 (28.2–34.8)	26	11,455	11,314	23 (15.0–33.7)	23	5,972	5,830	39.4 (25.0–59.2)
Year of surgery												
2011	22	20,238	19,986	11.0 (6.9 16.7)	1	2,225	2,201	4.5 (0.1–25.3)	4	1,101	1,075	37.2 (10.1–95.2)
2012	40	21,580	21,256	18.8 (13.4– 25.6)	3	2,270	2,241	13.4 (2.8–39.1)	5	1,132	1,109	45.1 (14.6–105)
2013	53	22,418	22,080	24.0 (18.0–31.4)	2	2,225	2,203	9.1 (1.1–32.8)	4	1,140	1,117	35.8 (9.8–91.7)
2014	94	24,232	23,861	39.4 (31.8–48.2)	10	2,353	2,324	43.0 (20.6–79.1)	4	1,262	1,231	32.5 (8.9–83.2)
2015	141	24,817	24,379	57.8 (48.7–68.2)	10	2,382	2,344	42.7 (20.5–78.4)	6	1,337	1,299	46.2 (17.0–101)
Sex												
Male	173	37,855	37,115	46.6 (39.9–54.1)	17	3,920	3,856	44.1 (25.7–70.6)	12	2,065	2,003	59.9 (31 104.7)
Female	175	75,122	74,144	23.6 (20.2 27.4)	9	7,526	7,448	12.1 (5.5–22.9)	11	3,898	3,819	28.8 (14.4–51.5)
Missing	2	308	304	65.9 (8.0–238)	0	9	9	0 (0–4087)	0	9	9	0 (0–4087)
Age												
Missing	0	178	176	0 (0–209)	0	4	4	0 (0–9194)	0	8	7	0 (0–5047)
< 60	68	19,092	18,859	36.1 (28.0–45.7)	8	2,172	2,156	37.1 (16.0–73.1)	5	1,127	1,110	45.0 (14.6–105)
60–65	58	19,156	18,936	30.6 (23.3–39.6)	4	1,889	1,871	21.4 (5.8–54.7)	8	986	968	82.7 (35.7–163)
66–70	79	21,791	21,536	36.7 (29.0–45.7)	7	2,105	2,084	33.6 (13.5–69.2)	3	1,137	1,114	26.9 (5.6–78.7)
71–75	55	21,076	20,809	26.4 (19.9–34.4)	2	2,143	2,122	9.4 (1.1–34.1)	3	1,120	1,094	27.4 (5.7–80.1)
76–80	56	18,104	17,790	31.5 (23.8–40.9)	2	1,788	1,763	11.3 (1.4–41.0)	3	881	848	35.4 (7.3–103)
> 80	34	13,888	13,457	25.3 (17.5–35.3)	3	1,354	1,313	22.8 (4.7–66.8)	1	713	689	14.5 (0.4–80.9)
BMI												
Missing	91	57,099	56,295	16.2 (13.0–19.8)	6	6,344	6,268	9.6 (3.5–20.8)	10	2,981	2,922	34.2 (16.4–62.9)
< 18.5	3	477	460	65.2 (13.4–190)	0	40	36	0 (0–1011)	0	41	37	0 (0–992)
18.5–24.9	36	17,623	17,346	20.8 (14.5–28.7)	5	1,639	1,618	30.9 (10–72.1)	3	1,013	990	30.3 (6.3–88.6)
25–29.9	106	24,280	23,916	44.3 (36.3–53.6)	9	2,201	2,175	41.4 (18.9–78.6)	4	1,208	1,172	34.1 (9.3–87.4)
30–39.9	100	13,109	12,872	77.7 (63.2–94.5)	5	1,153	1,140	43.9 (14.2–102)	6	694	675	88.8 (32.6–193)
≥ 40	14	697	673	208 (114–349)	1	78	76	131 (3.3–733)	0	35	34	0 (0–1082)
ASA												
Missing	5	1,138	1,118	44.7 (14.5–104.3)	0	333	332	0 (0–111)	1	88	83	121 (3.1–672)
I	49	23,602	23,411	20.9 (15.5–27.7)	4	2,428	2,411	16.6 (4.5–42.5)	3	1,177	1,170	25.6 (5.3–75)
II	217	73,177	72,235	30.0 (26.2–34.3)	16	7,269	7,198	22.2 (12.7–36.1)	13	3,743	3,669	35.4 (18.9–60.6)
III–IV	79	15,368	14797	53.4 (42.3–66.5)	6	1425	1372	43.7 (16.0–95.2)	6	964	909	66.0 (24.2–144)
Surgical indication												
Missing	5	978	952	52.5 (17.0–123)	0	156	153	0 (0–241)	0	55	49	0 (0–754)
Osteoarthritis	297	98,160	96,929	30.6 (27.3–34.3)	18	9,761	9,666	18.6 (11.0–29.4)	19	5,001	4,910	38.7 (23.3–60.4)
Trauma	22	6,718	6,424	34.2 (21.5–51.9)	5	689	662	75.6 (24.5–176)	2	415	384	52.1 (6.3–188)
Other indication	26	7,429	7,257	35.8 (23.4–52.5)	3	849	833	36.0 (7.4–105)	2	501	488	41.0 (5.0–148)
Surgical approach												
Missing	3	667	656	45.8 (9.4–133.7)	0	109	107	0 (0–345)	0	22	22	0 (0–1682)
Posterolateral	250	69,871	68,737	36.4 (32.0–41.2)	18	7,675	7,580	23.7 (14.1–37.5)	16	3,311	3,234	49.5 (28.3–80.3)
Anterior	23	12,948	12,822	17.9 (11.4–26.9)	2	288	287	69.7 (8.4–252)	0	612	598	0 (0–61.7)
Anterolateral	74	2,9799	29348	25.2 (19.8–31.7)	6	3383	3340	18 (6.6–39.1)	7	2027	1977	35.4 (14.2–73.0)
Fixation												
Missing	1	469	460	21.7 (0.6–121)	0	34	34	0 (0–1100)	0	10	8	0 (0–4543)
Cemented	101	29,191	28,638	35.3 (28.7–42.9)	18	5,685	5,606	32.1 (19.0–50.7)	7	1,293	1,254	55.8 (22.5–115)
Hybrid	43	9,829	9,654	44.5 (32.2–60.0)	1	1,685	1,665	6.0 (0.2–33.5)	4	921	903	44.3 (12.1–113)
Uncemented	205	73,796	72,811	28.2 (24.4–32.3)	7	4,051	4,008	17.5 (7.0–36.0)	12	3,748	3,665	32.7 (16.9–57.2)
Bearings surface												
Missing	34	10,459	10,227	33.2 (23.0–46.5)	0	603	590	0 (0–62.6)	0	129	125	0 (0–296)
Ceramic on PE	181	56,009	55,231	32.8 (28.2–37.9)	11	5,943	5,876	18.7 (9.3–33.5)	11	1,587	1,557	70.6 (35.3–126)
Metal on PE	110	31,121	30,585	36.0 (29.6–43.3)	14	2,721	2,683	52.2 (28.5–87.5)	7	2,809	2,729	25.6 (10.3–52.8)
Ceramic on ceramic	10	8,231	8,159	12.3 (5.9–22.5)	1	2,069	2,046	4.9 (0.1–27.2)	2	818	797	25.1 (3.0–90.7)
Zirconium on PE	14	6,486	6,395	21.9 (12.0–36.7)				(–)	0	507	503	0 (0–73.3)
Metal on metal	1	979	966	10.4 (0.3–57.7)	0	119	118	0 (0–312)	3	122	119	252 (51.9–735)

Appendix Table 3. Revision rates for infection per 10,000 person-years (PYs) after knee replacement by antibiotics regimen

	Cefazolin multi-dose				Cefazolin single -dose				Cefuroxime multi-dose			
Factor	Revisions	n	PYs	Rate (95% CI)	Revisions	n	PYs	Rate (95% CI)	Revisions	n	PYs	Rate (95% CI)
Overall	260	96,791	96,237	27 (23.8–30.5)	24	9,880	9,826	24.4 (15.6–36.3)	19	4,796	4,767	39.9 (24–62.2)
Year of surgery												
2011	19	16,735	16,652	11.4 (6.9–17.8)	2	1,785	1,775	11.3 (1.4–40.7)	2	927	924	21.7 (2.6–78.2)
2012	48	18,740	18,643	25.7 (19.0–34.1)	1	1,925	1,915	5.2 (0.1–29.1)	2	948	945	21.2 (2.6–76.5)
2013	55	19,288	19,162	28.7 (21.6–37.4)	3	1,911	1,909	15.7 (3.2–45.9)	1	977	970	10.3 (0.3–57.4)
2014	53	20,920	20,814	25.5 (19.1–33.3)	6	2,170	2,154	27.9 (10.2–60.6)	6	1,018	1,008	59.5 (21.8–130)
2015	85	21,108	20,966	40.5 (32.4–50.1)	12	2,089	2,073	57.9 (29.9–101)	8	926	920	87.0 (37.5–171)
Sex												
Male	143	33,501	33,244	43.0 (36.3–50.7)	16	3,317	3,293	48.6 (27.8–78.9)	7	1,539	1,532	45.7 (18.4–94.1)
Female	115	62,932	62,638	18.4 (15.2–22.0)	8	6,558	6,528	12.3 (5.3–24.1)	12	3,250	3,228	37.2 (19.2–64.9)
Missing	2	358	355	56.3 (6.8–203)	0	5	5	0 (0–7356)	0	7	7	0 (0–5254)
Age												
Missing	1	152	150	66.8 (1.7–372)	0	4	4	0 (0–9195)	0	7	7	0 (0–5254)
< 60	47	17,104	17,038	27.6 (20.3–36.7)	5	1,726	1,721	29.1 (9.4–67.8)	6	769	761	78.8 (28.9 172)
60–65	55	19,282	19,216	28.6 (21.6–37.3)	3	1,951	1,949	15.4 (3.2–45)	6	948	942	63.7 (23.4 139)
66–70	53	19,483	19,414	27.3 (20.4–35.7)	7	1,905	1,899	36.9 (14.8–76)	2	983	980	20.4 (2.5–73.7)
71–75	43	17,982	17,892	24.0 (17.4–32.4)	3	1,893	1,885	15.9 (3.3–46.5)	2	938	938	21.3 (2.6–77)
76–80	37	13,566	13,455	27.5 (19.4–37.9)	4	1,367	1,351	29.6 (8.1–75.8)	1	664	661	15.1 (0.4–84.3)
> 80	24	9,222	9,072	26.5 (17–39.4)	2	1,034	1,017	19.7 (2.4–71)	2	487	479	41.8 (5.1 150.9)
BMI												
Missing	97	48,461	48,194	20.1 (16.3–24.6)	6	5,458	5,435	11 (4.1–24.0)	4	2,518	2,508	16.0 (4.3–40.8)
< 18.5	0	96	95	0 (0–387)	1	15	15	673 (17.1–3754)	0	2	2	0 (0–18389)
18.5–24.9	20	7,664	7,601	26.3 (16.1–40.6)	1	727	720	13.9 (0.4–77.4)	2	370	369	54.3 (6.6–196)
25–29.9	74	19,919	19,791	37.4 (29.4–46.9)	8	1,815	1801	44.4 (19.2–87.5)	6	890	882	68.0 (25.0–148)
30–39.9	60	18,701	18,620	32.2 (24.6–41.5)	7	1,687	1679	41.7 (16.8–85.9)	7	931	925	75.7 (30.4–156)
≥ 40	9	1,950	1,935	46.5 (21.3–88.3)	1	178	177	56.6 (1.4–315)	0	85	83	0 (0–446)
ASA												
Missing	3	1,350	1,340	22.4 (4.6–65.4)	0	314	314	0 (0–118)	0	78	78	0 (0–472)
I	41	16,189	16,138	25.4 (18.2–34.5)	5	1,509	1,503	33.3 (10.8–77.6)	4	636	633	63.2 (17.2 162)
II	167	65,977	65,649	25.4 (21.7 29.6)	13	6,749	6,722	19.3 (10.3–33.1)	12	3,276	3,259	36.8 (19–64.3)
III–IV	49	13,275	13,109	37.4 (27.7–49.4)	6	1,308	1,288	46.6 (17.1 101.4)	3	806	798	37.6 (7.8 109.9)
Surgical indication												
Osteoarthritis	234	92,427	91,909	25.5 (22.3–28.9)	19	9,232	9,183	20.7 (12.5–32.3)	19	4,548	4,521	42.0 (25.3–65.6)
Trauma	11	1,495	1,480	74.3 (37.1–133)	0	135	135	0 (0–273)	0	105	104	0 (0–355)
Rheumatic	7	1,380	1,369	51.1 (20.6–105)	1	186	185	54.0 (1.4–301)	0	65	65	0 (0–567)
Other indication	8	1,489	1,479	54.1 (23.4–107)	4	327	323	124 (33.7–317)	0	78	78	0 (0–476)
Surgical approach												
Missing	3	1,241	1,237	24.3 (5.0–70.9)	0	89	89	0 (0–413)	0	19	19	0 (0–1936)
Medial parapatellar	243	90,617	90,100	27.0 (23.7–30.6)	21	9,628	9,578	21.9 (13.6–33.5)	17	4,169	4,144	41.0 (23.9–65.7)
Mid-/sub- vastus	9	3,715	3,698	24.3 (11.1–46.2)	1	90	89	112 (2.8–626)	1	592	589	17.0 (0.4–94.7)
Other approach	5	1,218	1,203	41.6 (13.5–97)	2	73	70	285 (34.5–1030)	1	16	16	624 (15.8–3479)
Fixation												
Missing	1	882	880	11.4 (0.3–63.3)	0	42	42	0 (0–876)	0	20	20	0 (0–1839)
Cemented	247	86,406	85,905	28.8 (25.3–32.6)	21	9,202	9,154	22.9 (14.2–35.1)	19	4,763	4,735	40.1 (24.2–62.7)
Hybrid	3	5,145	5,113	5.9 (1.2–17.1)	0	16	16	0 (0–2351)	0	1	0	0 (0–13x10^6)
Uncemented	9	4,358	4,338	20.7 (9.5–39.4)	3	620	615	48.8 (10.1–143)	0	12	12	0 (0–3065)
